# The Role of Medication Data to Enhance the Prediction of Alzheimer's Progression Using Machine Learning

**DOI:** 10.1155/2021/8439655

**Published:** 2021-09-21

**Authors:** Shaker El-Sappagh, Tamer Abuhmed, Bader Alouffi, Radhya Sahal, Naglaa Abdelhade, Hager Saleh

**Affiliations:** ^1^Centro Singular de Investigacion en Tecnoloxias Intelixentes (CiTIUS), Universidade de Santiago de Compostela, Santiago de Compostela, Spain; ^2^College of Computing, Sungkyunkwan University, Seoul, Republic of Korea; ^3^Department of Computer Science, College of Computers and Information Technology, Taif University, P. O. Box 11099, Taif 21944, Saudi Arabia; ^4^Faculty of Computer Science and Engineering, Hodeidah University, Al Hudaydah, Yemen; ^5^Information Systems Department, Faculty of Computers and Information, Assiut University, Assiut, Egypt; ^6^Faculty of Computers and Artificial Intelligence, South Valley University, Hurghada, Egypt

## Abstract

Early detection of Alzheimer's disease (AD) progression is crucial for proper disease management. Most studies concentrate on neuroimaging data analysis of baseline visits only. They ignore the fact that AD is a chronic disease and patient's data are naturally longitudinal. In addition, there are no studies that examine the effect of dementia medicines on the behavior of the disease. In this paper, we propose a machine learning-based architecture for early progression detection of AD based on multimodal data of AD drugs and cognitive scores data. We compare the performance of five popular machine learning techniques including support vector machine, random forest, logistic regression, decision tree, and K-nearest neighbor to predict AD progression after 2.5 years. Extensive experiments are performed using an ADNI dataset of 1036 subjects. The cross-validation performance of most algorithms has been improved by fusing the drugs and cognitive scores data. The results indicate the important role of patient's taken drugs on the progression of AD disease.

## 1. Introduction

Alzheimer's disease (AD) is considered as one of the most severe diseases that destroy the brain (Zheng and Xu [1]). According to the Alzheimer's Association report by Huber-Carol et al. [2], more than sixty million people around the globe would suffer from AD in the next fifty years. Moreover, based on the report estimation, one person is affected by dementia every three seconds. Consequently, by 2050, the potential number is 152 million internationally [3]. As dementia has several stages, there is a stage called mild cognitive impairment (MCI) between healthy aging and AD. Most people with MCI are gradually advance to dementia within five years (Ye et al. [4]). In addition, MCI patients who are ranged between *d* 10% to 20% convert to AD per year as estimated by Qiu et al. [5]. Therefore, the early-stage discovery of AD could provide an opportunity for a treatment that slows down AD symptoms and improve the patient's life (Gray et al. [6]). The early identification of patients in whom AD and progressive MCI (pMCI) is converted from stable MCI (sMCI) is a complex problem because patients always have similar signs (Lee et al. [7]). Machine learning (ML) techniques is playing an essential role in many areas such as engineering, physics, mathematics, marketing, and computer science (Liu et al. [8, 9]). ML techniques have great potential to adopt with this medical challenge (Liu et al. [10]). As AD is considered as chronic disease, the collected patients' data are considered to be time-series and multimodal data. Furthermore, the AD patients' data is considered as heterogeneous based on the patients' profiles. Recently, several ML models such as K-nearest neighbor (KNN), support vector machine (SVM), multilayer perceptron (MLP), and logistic regression (LR) have been employed to classify a patient as cognitive normal (CN), MCI, or AD (Moradi et al. [11]; Park et al. [12]). These studies focus primarily on using single modalities including magnetic resonance imaging (MRI) (Liu et al. [10]), fluoro-deoxyglucose positron emission tomography (FDGPET) (Hinrichs et al. [13]), diffusion tensor imaging (DTI), and cerebrospinal fluid (CSF). However, using single modalities negatively affects models' performance because some useful additional information from various biomarker modalities is omitted (Ye et al. [4]). Some studies have investigated the combination of multiple modalities for AD classification, and they achieved better performance compared to methods based on single modalities (Gray et al. [6], Zhang et al. [14]). In this context, Wee et al. [15] used both DTI and MRI to identify ten patients with MCI from 17 matched CN patients. The accuracy is increased by 7.4% better than using the single-modality-based method. Bouwman et al. [16] diagnosed CN patients from MCI using two modalities: MRI and CSF. For predicting cognitive loss in MCI, Fellgiebel et al. [17] used PET and CSF to predict cognitive loss in MCI. Zhang et al. [14] classified AD and MCI from CN using integration between three modalities: MRI, FDGPET, and CSF. Gray et al. [6] applied a random forest (RF) algorithm to four modalities: genetics, MRI, CSF, and FDGPET to classify AD versus MCI versus CN. In the other hand, there are some works that used time-series approaches to detect AD progression. The authors in Moradi et al. [11] used semisupervised learning to predict MCI-to-AD conversion between one to three years using MRI modality.

The authors in El-Sappagh et al. [18] used ensemble machine learning classifiers based on RF for the two layers, utilizing multimodal AD datasets. Venugopalan et al. [19] used different models including, SVM, DT, RF, and KNN, to early detect the AD stage. In addition, they demonstrated multimodality data and single-modality models. Moore et al. [20] studied the relationship between pairs of data points at various time separations using RF. In addition, they used three modalities: demographic, physical, and cognitive data.

Model performance is improved using time-series data with multimodel consideration for AD progression detection. The resulting models are expected to be more stable and medically acceptable because they mimic the real procedures followed by medical experts. In addition to MRI, PET, and CSF, there is a crucial data source, which has not been studied in the literature of AD. This data source is dementia medications, which are taken during patient's observation period. The drugs contains of chemical substances which are accumulated in the body in some forms, which increases the probability of disease progression, or the drugs could help to improve the patient conditions, which decreases the probability of disease progression. Thus, it is necessary to study the impact of these drugs on the disease's progression (Zimmerman [21]). Furthermore, there is no study in the literature that discussed this issue. In this work, we have provided an ML-based model to predict AD progression after 2.5 years. In doing so, we have implemented and tested a set of ML techniques according to the patient multimodal time-series data. The study is based on the cognitive score and Alzheimer's medication (AM) data. For every patient, these modalities are collected for 1.5 years (baseline, month-6, month-12, and month-18) and used to predict the patient's state at month 48. We used the ADNI dataset. ADNI is real clinical data, so our results have potential practical applications. Extensive experiments have been performed, and AM data showed the superiority of improving the CV performance of most algorithms. All models have been optimized using the grid search technique. Furthermore, the effect of the feature selection process on the model's performance has been studied.

The rest of this paper is structured as follows: Section 2 presents the architecture of the proposed system of predicting Alzheimer's progression.  Section 3 describes the experimental results. Finally, the paper is concluded in Section 4.

## 2. The Proposed System of Predicting Alzheimer's Progression

**Figure 1:**
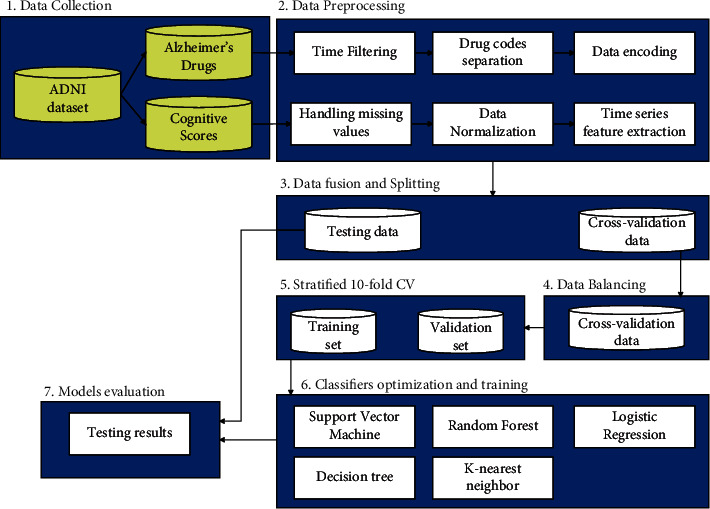
The architecture of the proposed system of predicting Alzheimer's progression.

The proposed system of predicting Alzheimer's progression is described in Figure 1. It consists of the following steps: data collection, data preprocessing, data fusion and splitting, data balancing, classifiers optimization and training, and models evaluation. Each step of the proposed system is described in detail in the following subsections.

### 2.1. Data Collection

Data used in this work was collected from the Alzheimer's disease neuroimaging initiative (ADNI) database disease neuroimaging initiative [22]. Over 57 sites in the United States and Canada have enrolled subjects El-Sappagh et al. [18]. The study was carried out in accordance with GCP principles, the Declaration of Helsinki, and US 21 CFR part 50 —Protection of Human Subjects—and part 56 —Institutional Review Boards. Subjects were willing and able to participate in test procedures such as neuroimaging and follow-up, and they gave written informed consent. All data are open to the public at disease neuroimaging initiative [22]. The collected dataset has 1036 subjects categorized into four groups, as shown in Table 1. The study is based on two time-series modalities of the cognitive score (CS) and Alzheimer's medication (AM). The CS dataset includes eight features: CDRSB, GDTOTAL, FAQ, ADAS 13, CDG, MMSE, MOCA, and NPISCORE. Based on the ADNI dataset, we designed a drug dataset that includes nine features: antidepressant, Cognex, Aricept, Namenda, Exelon, Razadyne, Other, and None. These drugs are sorted according to their popularity in our dataset as Aricept, Namenda, antidepression, Exelon, and Cognex (42.18%, 25.77%, 23.84%, 6.18%, and 0.09%, respectively). Mostly the CN (85.94%) patients did not take any drugs. As a result, we removed this class from the dataset. All datasets have 787 patients and three classes (sMCI, pMCI, and AD). Table 1 shows the patients' demographics.

#### 2.1.1. Data Preprocessing

**Figure 2:**
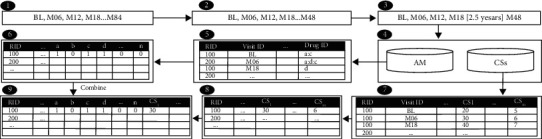
Data fusion steps.

We prepare both the drugs and cognitive scores datasets that we collected from the ADNI dataset, as shown in Figure 2. The drug dataset has several preprocessing steps:

  Time filtering: In this phase, we filtered data of four visits, that is, the first four visits (bl, M06, M12, M18) denoted to baseline, month 6, month 12, and month 18, respectively. These visits data are exploited with and without drug data to explore the effects of drugs on predicting an AD patient's progression after 2.5 years (at month 48).
  Code separation: The drug dataset includes a column containing multiple values and delimiter “:” that separates values. We separate the row into two multirows using “:” delimiter. The Cognex feature has been removed because only 0.09% of the patients used it.
  Data encoding: The dataset includes a column with names of patients' drugs; we split each drug's name and create a new dataset that includes nine columns. The names of the drugs are listed in these columns. Each column has a binary value (i.e., 0 or 1) indicating whether or not the patient is taking the drug.
  Aggregation: The last dataset includes multiple rows of each patient. We convert multiple rows of each patient into one row by grouping rows using the RID column and get max value for each column.

The preprocessing of the CS dataset has been done as follows:

  The randomness of the data has been checked, and the data are missing at random.
  To minimize the negative effect of missing data on our dataset, any case with missing baseline scores or features with missing values of more than 30% was deleted. We used the forward filling technique to handle missing time-series data, where the previous values were used if the diagnosis was not changed for a time step.
  Data normalization for CS data has been done using the z-score method. Fourth, aggregated features from the four historical time steps were collected to summarize time-series data. We took the average of each CS for each patient.

#### 2.1.2. Dataset Splitting

Given the two collected features sets (i.e., CS, AM), three datasets are created: (1) CS features only dataset, (2) AM features dataset, and (3) CS-AM dataset, where the CS and AM modalities are fused. The stratified method is used to split each dataset into 90% of the dataset as the training set, and 10% of the dataset as the unseen test set. The Ml models were trained and optimized using the training set; the ML models were evaluated using the unseen test set.

#### 2.1.3. Dataset Balancing

Biased models are always the outcome of unbalanced datasets. The synthetic minority oversampling approach (SMOTE) proposed by Chawla et al. [23] was used to handle the class imbalance to avoid the biased models. The SMOTE is applied to only the training set.

#### 2.1.4. Classifiers Optimization and Training

The optimal values of hyperparameters of the ML models were selected using the grid search approach with stratified 10-fold CV. The five were applied to each dataset:

  Support vector machine (SVM) is a supervised learning approach that analyzes data for classification or regression. The SVM is a discriminative algorithm that is formalized by an optimum hyperplane. It generates an optimal hyperplane result, which classifies unknown instances, and datasets that support the hyperplane are referred to as support vectors. However, selecting the optimum hyperplane is tough since it must be noise-free and accurate in its generalization of data sets. SVM is attempting to discover an optimum hyperplane that delivers a significant minimum distance to the trained data set.
  Decision tree (DT) by Sweety and Jiji [24] is one of the most widely used machine learning classifiers. It is pretty trendy because it can be customized to nearly all kinds of data types. It is a supervised learning technique that partitions training data into smaller chunks to extract patterns for classification. The knowledge is then shown as a tree, which is easy to understand. The decision model is constructed from the top-down of the tree structure, beginning with the (top) root node. The root nodes are significant predictors, while the leaf nodes have a final classification.
  K-nearest neighbor (KNN) is a type of supervised algorithm. A KNN algorithm attempts to locate the pattern space for the *k* instances of training that are similar in new instances when analyzing testing data. KNN classifier may be appropriate for the dependent variable, covering two principles: low risk, medium risk, and high risk. Moreover, the KNN classification needs the same number of bad and good sample examples for better performance. The selection of *k* also fulfills the KNN process performance.
  Random forest (RF) by Alickovic et al. [25] is a machine learning classifier based on trees that leverages the power of multiple decision trees for making decisions. RF is made up of several decision trees, each of which chooses its separation features from a bootstrap training set. RF offers several advantages: the approach of classification is exact, quick, and noise-resistant. In RF, random selection and bagging features are merged. The values of independently sampled random vectors are influenced by every tree in the forest and have the same distribution as every other tree.
  Logistic regression (LR) Mirzaei et al. [26] is a supervised machine learning classifier that predicts the likelihood of a target variable. It is a multivariate technique that seeks to create functional relationships between numerous predictor variables and a single output. In most situations, the LR output variable is categorical because it can only be assigned to a limited number of classes. LR is a powerful ML algorithm because it can generate probabilities and categorize new data using discrete and continuous datasets.

#### 2.1.5. Evaluation Metrics

Models are evaluated using four standard metrics: accuracy, precision, recall, and F1-score, where TP stands for true positive, TN for true negative, FP for false positive, and FN for false negative, as shown in equations (1)–(4).

(1) $$\begin{array}{c} \text{Accuracy}=\frac{\text{TP}+\text{TN}}{\text{TP}+\text{FP}+\text{TN}+\text{FN}}, \end{array}$$

(2) $$\begin{array}{c} \text{Precision}=\frac{\text{TP}}{\text{TP}+\text{FP}}, \end{array}$$

(3) $$\begin{array}{c} \text{Recall}=\frac{\text{TP}}{\text{TP}+\text{FN}}, \end{array}$$

(4) $$\begin{array}{c} F1=\frac{2\cdot \text{precision}\cdot \text{recall}}{\text{precision}+\text{recall}}. \end{array}$$

## 3. Results

The Python 3.7.3 distributed in Anaconda 4.7.7 (64-bit) were used to run the experiment. The models were implemented using the Scikit-Scikit-learn 0.20.0 library Pedregosa et al. [27] The performance of ML models: SVM, LR, KNN, DT, and RF were registered to three datasets: CS, AM, and AM-CS. Three experiments were conducted to obtain the results. Each conducted experiment has been repeated 6 times, and the average of accuracy, precision, recall, and F1-score was registered (where A: Accuracy, P: Precision, R: Recall, and F1: F1-score). In the first experiment, we initially aimed to evaluate the capability of the ML models to distinguish patients of AD, pMCI, and sMCI classes based on either cognitive scores or Alzheimer's medication. Then, we tried to answer the question: to what extent does the features infusion of the CS and AM affect the performance of the ML models? Table 2 presents the first experimental results. In the second experiment, we evaluated the effect of AM on detecting pMCI within MCI patients. The experiment tries to answer the question: to what extent does the AM-CS fusion con-tribute to the overall performance of the ML models within the MCI patients? Table 3 presents the second experimental results. The third experiment is similar to experiment 2; however, this experiment answers the question: to what extent does the AM-CS fusion con-tribute to the overall performance of the ML models between the MCI and AD patients? Table 4 presents the third experimental results. For the last two experiments, we try to evaluate the performance of the ML models for MCI patients, who have similar cognitive scores and Alzheimer's medication, and sMCI vs. AD patients, who have medically different cognitive scores and Alzheimer's medication.

### 3.1. Experiment 1: sMCI vs. pMCI vs. AD

Table 2 shows that the ML models achieved the best CV performance for the fused dataset. For example, the RF, DT, LR, SVM, and KNN models achieved an accuracy of 92.74%, 84.96%, 88.4%, 82.89%, and 82.43%, respectively. RF is an ensemble classifier, which could be the main reason for its high performance. For the testing performance, three out of the five ML models achieved the highest performance using the fused dataset with accuracies 88.54%, 85.42%, and 74.22% for RF, LR, and KNN models, respectively. This indicates the importance of the AM data for the AD progression detection task. Table 2 also shows that the AM features alone are insufficient and CS-based models can be improved by AM-CS fusion.

### 3.2. Experiment 2: sMCI vs. pMCI

The results of this experiment as shown in Table 3 assert the crucial role of AM-CS fusion to enhance the ML model's performance. For the CV results, the RF, DT, LR, and SVM models with the AM-CS dataset outperformed other models with accuracies 87.90%, 89.54%, 87.07%, and 87.10%, respectively. Besides, testing results of these four ML models show an improvement using the AM-CS dataset with accuracies 85.11%, 89.36%, 87.23%, and 86.57% for RF, DT, LR, and SVM models, respectively. These models achieved testing AUC of 0.878, 0.815, 0.910, and 0.897 for RF, DT, LR, and SVM models, respectively. The results of ML models based on AM dataset alone achieved better performance than recent studies such as Ye et al. [4]. For example, the KNN and SVM models achieved testing accuracies 75.69% and 69.68%. These results are better than the Yao et al. model, which achieved an accuracy of 68.98% based on neuroimaging data.

### 3.3. Experiment 3: sMCI vs. AD

Similar to experiment 2, the results of this experiment assert the importance of AM-CS fusion to improve the detection of AD patients from MCI cases. Moreover, the performance of this experiment is better than the previous two experiments because all ML models can easily separate both sMCI and AD cases. For the CV results, the RF, DT, LR, and SVM models with AM-CS dataset outperformed other models with accuracies 94.96%, 93.66%, 94.12%, and 94.50%, respectively. Besides, testing results of these four ML models show an improvement using the AM-CS dataset with accuracies 94.82%, 95.95%, 90.09%, and 94.82% for RF, LR, SVM, and KNN models, respectively. These models achieved testing AUC of 0.941, 0.955, 0.897, and 0.941 for RF, LR, SVM, and KNN models, respectively. All the experiments confirmed our hypothesis that AM-CS fusion has a positive effect on the performance of progression detection problem in Alzheimer's disease.

## 4. Conclusion

This paper studies the role of dementia drugs in improving the progression detection for AD patients based on multimodal time-series data. The algorithm is based on the patient's four-time-step time-series data and can predict AD within 2.5 years of M18. The model is based on the early merging of time-series modalities from CS and AM. We have optimized and tested five ML models using the real-world ADNI dataset. The results showed the crucial role of drugs features to enhance the performance of these ML models. In the future, we will extend this work by studying the interpretability features of these models.

## Figures and Tables

**Table 1:** Patients' statistics at baseline.

	sMCI (*n* = 363)	pMCI (*n* = 106)	AD (*n* = 318)	Combined (*n* = 1036)
Sex (M/F)	210/153	44/62	142/176	483/553
Age (years)	72.92 ± 07.76	73.89 ± 06.84	75.01 ± 07.81	73.82 ± 07.18
Education	15.80 ± 02.97	16.13 ± 02.71	15.13 ± 02.98	15.85 ± 02.90
FAQ	02.64 ± 03.31	07.63 ± 04.49	16.42 ± 06.59	06.81 ± 08.01
MMSE	27.62 ± 01.95	25.46 ± 01.84	20.95 ± 03.95	25.66 ± 04.17
MoCA	22.96 ± 02.21	20.69 ± 01.84	17.11 ± 03.43	21.56 ± 04.14
APOE4	00.51 ± 00.66	00.85 ± 00.71	00.85 ± 00.71	00.56 ± 00.67
ADAS 13	14.69 ± 06.71	22.69 ± 05.29	33.59 ± 09.39	19.73 ± 12.24
ADAS 11	09.18 ± 04.47	14.25 ± 03.90	22.82 ± 08.09	12.96 ± 08.88
CDRSB	01.42 ± 00.79	02.99 ± 01.19	05.99 ± 02.49	02.67 ± 02.79

*∗* Data are mean ± standard deviation.

**Table 2:** The performance for the AD vs. pMCI vs sMCI task.

		Testing performance				Cross-validation performance			
Model	Dataset	*A*	*P*	*R*	*F*1	*A*	*P*	*R*	*F*1
RF	CS	87.76	87.76	87.79	87.76	90.35 ± 2.9	90.49 ± 2.8	90.47 ± 2.7	90.28 ± 2.5
	AM	68.05	67.47	68.82	68.05	58.39 ± 4.1	58.3 ± 4.26	60.74 ± 4.3	58.27 ± 4.2
	AM-CS	88.54	88.51	88.92	88.54	92.74 ± 3.1	92.87 ± 3.1	93.03 ± 2.6	93.21 ± 3.3

DT	CS	90.89	90.94	91.15	90.89	83.08 ± 4.9	83.08 ± 4.9	83.49 ± 4.9	83.13 ± 4.8
	AM	64.81	64.64	69.15	64.81	53.23 ± 4.3	53.23 ± 4.6	55.27 ± 4.7	53.21 ± 4.2
	AM-CS	89.32	89.30	89.74	89.32	84.96 ± 3.7	84.95 ± 3.7	85.61 ± 3.5	85.15 ± 3.7

LR	CS	76.85	77.15	78.34	76.85	79.85 ± 4.4	79.96 ± 4.3	80.61 ± 4.2	79.85 ± 4.4
	AM	53.24	51.32	52.76	53.24	55.39 ± 4.6	54.64 ± 4.6	54.7 ± 4.71	55.39 ± 4.6
	AM-CS	85.42	85.40	85.64	85.42	88.40 ± 3.3	88.36 ± 3.3	88.91 ± 3.1	88.4 ± 3.28

SVM	CS	81.02	81.11	81.40	81.02	79.95 ± 3.9	79.93 ± 4.0	80.7 ± 3.96	79.95 ± 3.9
	AM	58.33	57.05	58.85	58.33	55.35 ± 4.5	55.52 ± 4.4	56.54 ± 4.3	55.35 ± 4.5
	AM-CS	76.39	76.55	77.04	76.39	82.89 ± 3.1	82.78 ± 3.1	83.18 ± 3.1	82.89 ± 3.1

KNN	CS	73.61	74.31	76.53	73.61	76.32 ± 3.7	76.8 ± 3.68	80.52 ± 3.4	76.32 ± 3.7
	AM	48.00	42.67	45.20	48.00	52.31 ± 3.2	43.13 ± 3.4	39.62 ± 7.5	52.31 ± 3.2
	AM-CS	74.22	72.37	82.99	74.22	82.43 ± 4.2	82.11 ± 4.4	84.62 ± 3.8	82.43 ± 4.2

**Table 3:** The performance for the sMCI vs pMCI task.

		Testing performance				Cross-validation performance			
Model	Dataset	*A*	*P*	*R*	*F*1	*A*	*P*	*R*	*F*1
RF	CS	82.98	63.64	63.64	63.64	87.76 ± 4.0	88.56 ± 5.3	87.14 ± 6.3	87.66 ± 4.0
	AM	59.57	33.33	72.73	45.71	73.56 ± 5.2	70.70 ± 5.1	81.05 ± 7.9	75.37 ± 4.8
	AM-CS	85.11	66.67	72.73	69.57	87.90 ± 4.1	87.47 ± 4.8	88.68 ± 6.7	87.96 ± 4.2

DT	CS	87.16	75.00	81.82	78.26	89.30 ± 3.7	88.37 ± 4.7	88.97 ± 4.7	88.58 ± 3.6
	AM	55.32	40.77	72.73	52.25	72.79 ± 5.4	69.71 ± 4.4	81.03 ± 8.8	74.79 ± 5.2
	AM-CS	89.36	80.00	72.73	76.19	89.54 ± 3.6	89.34 ± 5.1	89.58 ± 6.9	89.27 ± 3.9

LR	CS	80.85	58.33	63.64	60.87	84.86 ± 4.0	86.86 ± 6.1	82.55 ± 4.6	84.53 ± 3.9
	AM	61.70	46.78	72.73	56.93	67.59 ± 4.6	67.68 ± 4.6	67.93 ± 5.4	67.68 ± 4.4
	AM-CS	87.23	76.47	77.47	76.96	87.07 ± 4.1	87.42 ± 4.9	86.90 ± 6.6	86.99 ± 4.3

SVM	CS	83.56	83.52	84.01	83.56	83.95 ± 4.5	83.86 ± 4.6	84.61 ± 4.4	83.95 ± 4.5
	AM	69.68	69.47	70.21	69.68	67.51 ± 4.0	67.07 ± 4.1	68.55 ± 4.5	67.51 ± 4.0
	AM-CS	86.57	86.57	86.65	86.57	87.10 ± 4.7	87.06 ± 4.7	87.48 ± 4.6	87.1 ± 4.72

KNN	CS	81.94	81.51	85.23	81.94	83.41 ± 3.6	82.99 ± 3.9	86.66 ± 3.0	83.41 ± 3.6
	AM	75.69	74.90	79.45	75.69	66.39 ± 6.7	65.46 ± 7.8	67.71 ± 6.6	66.39 ± 6.7
	AM-CS	75.23	74.92	76.50	75.23	79.62 ± 4.4	79.44 ± 4.5	80.59 ± 4.4	79.62 ± 4.9

**Table 4:** The performance for the AD vs sMCI task.

		Testing performance				Cross-validation performance			
Model	Dataset	*A*	*P*	*R*	*F*1	*A*	*P*	*R*	*F*1
RF	CS	94.14	94.14	94.24	94.14	94.76 ± 2.7	94.66 ± 2.9	95.1 ± 2.65	94.71 ± 2.8
	AM	77.48	77.43	77.76	77.48	77.12 ± 4.9	76.8 ± 5.11	78.59 ± 4.8	77.46 ± 5.1
	AM-CS	94.82	94.82	94.83	94.82	94.96 ± 2.4	94.96 ± 2.5	95.20 ± 2.3	94.91 ± 2.4

DT	CS	88.52	88.50	88.66	88.52	93.02 ± 2.7	93.01 ± 2.7	93.36 ± 2.5	93.08 ± 2.7
	AM	73.42	73.22	74.18	73.42	75.76 ± 5.1	75.18 ± 5.2	78.42 ± 5.4	75.76 ± 5.1
	AM-CS	84.91	84.90	85.05	84.91	93.66 ± 3.0	93.76 ± 2.8	93.83 ± 2.7	93.69 ± 2.9

LR	CS	92.34	92.34	92.40	92.34	94.05 ± 2.4	94.5 ± 2.39	94.64 ± 2.3	94.05 ± 2.4
	AM	72.30	71.92	73.54	72.30	78.99 ± 4.2	78.8 ± 4.32	80.02 ± 4.1	78.99 ± 4.2
	AM-CS	95.95	95.95	95.98	95.95	94.12 ± 2.8	94.11 ± 2.8	94.27 ± 2.7	94.12 ± 2.8

SVM	CS	89.86	89.86	89.89	89.86	94.19 ± 3.3	94.18 ± 3.3	94.36 ± 3.2	94.19 ± 3.3
	AM	75.23	75.22	75.24	75.23	77.82 ± 5.9	77.44 ± 6.1	79.5 ± 5.77	77.82 ± 5.9
	AM-CS	90.09	90.09	90.14	90.09	94.50 ± 2.6	94.5 ± 2.61	94.64 ± 2.5	94.5 ± 2.6

KNN	CS	92.79	92.79	92.81	92.79	94.17 ± 2.9	94.16 ± 2.9	94.29 ± 2.9	94.17 ± 2.9
	AM	70.27	70.04	70.94	70.27	78.10 ± 4.0	77.82 ± 4.1	79.46 ± 3.8	78.10 ± 4.0
	AM-CS	94.82	94.82	94.94	94.82	94.00 ± 2.8	93.99 ± 2.8	94.14 ± 2.8	94.0 ± 2.86

## Data Availability

The data were collected from the ADNI (http://adni.loni.usc.edu/). The ADNI data had previously been gathered from 50 different study sites. Requests for data access should be made to http://adni.loni.usc.edu/data-sampl es/access-data/.

## Supplementary Materials

Supplementary Materials The Supplementary file shows the ethics committees/institutional review boards that approved the ADNI study ()
